# A Review of the Distribution and Health Effect of Organophosphorus Flame Retardants in Indoor Environments

**DOI:** 10.3390/toxics12030195

**Published:** 2024-03-01

**Authors:** Xingwei Song, Sheng Zhu, Ling Hu, Xiaojia Chen, Jiaqi Zhang, Yi Liu, Qingwei Bu, Yuning Ma

**Affiliations:** 1 Jiangsu Environmental Monitoring Centre, Nanjing 210019, China; sxw@jshb.gov.cn (X.S.); hul@jshb.gov.cn (L.H.); 2 Quzhou Environmental Monitoring Centre, Quzhou 324000, China; wmaxwell@163.com; 3 School of Environment and Architecture, University of Shanghai for Science and Technology, Shanghai 200093, China; chenxiaojia@usst.edu.cn; 4 State Environmental Protection Key Laboratory of Formation and Prevention of Urban Air Pollution Complex, Shanghai Academy of Environment Sciences, Shanghai 200233, China; 5 School of Environmental Science and Engineering, Shanghai Jiao Tong University, Shanghai 200240, China; zjq560047@163.com; 6 Thomas Gosnell School of Life Sciences, Rochester Institution of Technology Rochester, New York, NY 14623, USA; yl5329@rit.edu; 7 School of Chemical & Environmental Engineering, China University of Mining & Technology, Beijing 100083, China; 8 College of Environmental and Resource Sciences, Zhejiang University, Hangzhou 310058, China

**Keywords:** organophosphorus flame retardants, indoor environment, human exposure, risk assessment

## Abstract

As a replacement for polybrominated diphenyl ethers (PBDEs), organophosphorus flame retardants (OPFRs) have been widely used and detected in different indoor environments all over the world. This paper comprehensively describes the concentration levels and distribution information of 11 kinds of OPFRs from 33 indoor dust and 10 air environments, from which TBOEP, TCIPP, and TDCIPP were observed to have higher concentrations in indoor environments. The ΣOPFRs displayed higher concentrations in indoor dust than in indoor air due to the higher molecular weight and vapor pressure of ΣOPFRs in building decoration materials, specifically for TCIPP and TDCIPP compounds. Considering that it is inevitable that people will be exposed to these chemicals in the indoor environments in which they work and live, we estimated their potential health risks through three human exposure pathways and found that the ingestion exposure to TBOEP for toddlers in Japan may reach up to 1270.80 ng/kg/day, which comprises a significant pathway compared to dermal contact and indoor air inhalation. Specifically, the combined total exposure to OPFRs by air inhalation, dust ingestion, and dermal contact was generally below the RfD values for both adults and toddlers, with a few notable higher exposures of some typical OPFRs.

## 1. Introduction

Flame retardants (FR) are chemicals that are added to various types of consumer goods and materials in order to prevent combustion and the spread of fire [1]. Since the 1970s, polybrominated diphenyl ethers (PBDEs) have been widely applied to consumer goods, as they have a strong flame-retardant property. However, some of them—such as penta-BDE, octa-BDE, and deca-BDE—were mostly banned by the European Union in 2008 due to their persistence, bioaccumulation, and toxicity. Then, as an alternative FR chemical, the emerging halogenated flame retardants (EHFR) were developed. Among the EHFRs, organophosphorus flame retardants (OPFRs), such as tris(2-chloroethyl) phosphate (TCEP) and tris(chloropropyl) phosphate (TCPP), are a group of alternative compounds that have been widely used in the global market to meet the needs of FR products [2].

Many kinds of indoor furniture, electric devices, and plastic products (e.g., sofas, furniture foam, carpets, mattresses, televisions, computers, mobile phones, upholstery, and textiles) may comprise and release a large amount of OPFRs [3,4]. For instance, the production of OPFRs in China reached 70,000 tons in 2007 [5], and the demand for emerging FRs has increased due to the restrictions on legacy PBDEs in the U.K. [6]. OPFRs frequently appear as additives rather than being chemically bonded to the final diverse products, which makes them easy to release [5,7,8,9]. OPFRs may leach by abrasion, volatilization, and directly contacted migration from the indoor products to their indoor environments [10]. The total concentrations of organic phosphate esters (OPEs) were 2–3 and 1–2 orders of magnitude higher than those of brominated flame retardants (BFRs) in air and dust/window films, respectively, in Canada, the USA, and the Czech Republic [11,12]. From 2012 to 2017, tributoxyethyl phosphate (TBEP) was the major component of the OPFR group in indoor dust environments in China [13,14,15]. The occurrence of OPFRs in indoor air and dust has been studied deeply in Europe [16,17]. In recent decades, global concern regarding the occurrence of OPFRs has been dramatically increasing, and OPFRs have been detected in more indoor sources and building materials such as beds, car seats, computer screens, TV sets, window film, and upholstery materials [18] Although outdoor pollution can affect indoor air quality, OPFRs are still present at significantly higher concentrations in closed environments than in outdoor areas [19]. Furthermore, some of them have low degradation potential and, as a result, they might be persistent in indoor environments [1]. Meanwhile, OPFRs have been found to be widely distributed in different environmental media (e.g., dust, air, water, soil), as well as in human serum and breast milk [1,20,21]. These studies reported the concentration profiles of OPFRs emitted from different indoor materials. As such, using methods such as principal components analysis (PCA) may help to accurately assess the indoor contamination source and patterns of OPFRs.

At present, many people usually spend over 20 h per day in indoor environments, making exposure to indoor pollution unavoidable in daily life [22]. Ingestion, inhalation, and dermal contact are three important absorptive routes for people to come into contact with these chemicals in indoor environments [23,24,25]. Regarding exposure to OPFRs, there are two routes: external exposure routes and internal exposure routes. External exposure includes exposures through dietary ingestion, dust ingestion, and air inhalation [26]. Daily intake of seafood and meat led to the exposure of humans to FRs through bioaccumulation, including OPFR metabolites such as DPP and BDCPP [27,28]. In addition, FRs include endocrine-disrupting chemicals (EDCs) that could lead to reproductive disorders and endocrine-related cancers and increase the prevalence of obesity. For instance, Cl-PFRs such as TCIPP, TDCIPP, and TCEP have been found to be carcinogenic and have adverse effects on human health, and TCrP and TNBP pose a potential threat as they may cause thyroid hormone disruption and reproductive toxicity [1,5,9]. Many studies have focused on the acute and long-term toxicity of OPFRs with respect to fish, rats, daphnia, and algae. Du et al. (2015) have found that Aryl-OPFRs, such as TPHP, could cause higher heart toxicity than alkyl-OPFRs by interfering with the expression of transcription regulators in zebrafish [29]. Toxicity data for OPFRs are incomplete, and most of them were obtained through animal studies, making it difficult to connect the toxicity associated with human exposure. Little is known about the combined environmental effect of OPFRs based on indoor concentration profiles and toxicity information. Hence, we plan to use meta-analysis to analyze the connections among their occurrence, concentration, and toxicity data.

This review summarizes the occurrences of and human exposure to OPFRs in indoor microenvironments and establishes an evaluative function that indicates the relationship among these parameters and variables. According to this evaluative function, the pollutant equivalency factors were calculated to conduct an overall assessment of the concentration level and toxicity of OPFRs. Then, several typical OPFRs measured in the indoor environments of different countries are further studied. Finally, the toxicity of PEFs is calculated, providing a basis for understanding which OPFRs should be targeted for control or intervention. To the best of our knowledge, this is the first review reporting an overall evaluation of OPFRs based on their global concentration levels and health effects in indoor environments.

## 2. Materials and Methods

### 2.1. Inclusion Criteria

This review aims to incorporate most of the studies that have been carried out describing the occurrence and distribution of organophosphorus flame retardants in indoor environments and their health effects, including human exposure and toxicity. Based on published literature from the Web of Science Core Collection and the Chinese Science Citation Database, studies were obtained by searching for “organophosphorus flame retardants” or “tris(chloropropyl) phosphate” in the title and “emerging pollutants”, “indoor environment”, or “phosphorus flame retardant” in the topic. Then, the irrelevant literature was eliminated by reading the titles and abstracts, and we supplemented our literature database by reading the references of the selected studies. The studies had to refer to organophosphorus flame retardants in indoor environments, including living houses and workplaces, through dust and air (as such, some studies referring to OPFRs in cars and building decoration materials were included). Studies reporting risk assessments of OPFRs regarding human exposure and toxicity were also included.

### 2.2. Search Strategy Description

**Figure 1:**
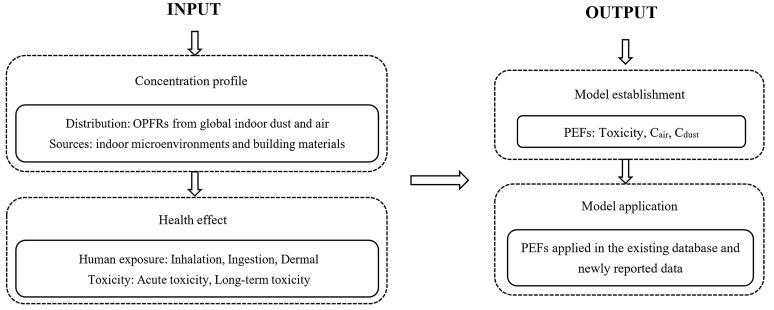
A schematic presentation of this review on OPFRs in indoor environments.

A comprehensive literature search was performed in the bibliographic databases Web of Science Core Collection and SCI Finder, covering studies published using the following keywords (and the combinations of them): (a) regarding organophosphorus flame retardants and indoor environments, “PFRs” (or “phosphorus flame retardant”) and “OPFRs” (or “organophosphorus flame retardants”) and “indoor” (or “indoor environments” or “indoor air” or “indoor dust” or “building materials”); (b) regarding OPFRs and human exposure to them, “organophosphorus flame retardants” and “human exposure” or “health effect” or “risk assessment”; (c) regarding OPFRs and their toxicity description, “organophosphorus flame retardants” and “toxicity”. Attention was paid to the data from different studies, as the conversion of units may be necessary for comparison. Table 1 provides the compound name, CAS number, abbreviation, molar mass, water solubility, vapor pressure, LogKoa, logKow, and instrumental identification ions or *m*/*z* of the OPFRs mentioned in this review. Figure 1 presents a schematic presentation of this review on OPFRs in indoor environments.

## 3. Concentration Profiles

### 3.1. Worldwide Distribution of OPFRs in Indoor Dust and Air

The processes that affect the fate of OPFRs in environments include sorption, volatilization, and biodegradation, which lead to different concentration profiles of organophosphorus flame retardants in indoor air and indoor dust according to the global region and microenvironment (Table 2 and Table 3). Vykoukalova et al. (2017) have found that there was no significant difference between the air in bedrooms and living rooms for 13 kinds of OPEs, while in the indoor dust, the concentrations of OPEs and BFRs were correlated [12]. For the dust in daycare centers, TBEP had the highest concentration (1,600,000 ng/g), while in workplaces, chlorinated organophosphate esters in the air (100 ng/m^3^) had a higher concentration in Stockholm, Sweden [32]. In the Japanese market, TPHP was dominant among 11 kinds of OPFRs, with concentrations ranging from 560 to 14,000,000 ng/g, while TBEP was not detected in an electronic appliance store [33]. However, for floor dust in Japan, TBEP was detected with the highest level in most samples [34], indicating that different indoor sources or locations would have compounds with different kinds and levels. In the Rhine/Main area in Germany, the ΣOPFRs median level in seven indoor microenvironments were as follows: private cars, 180.3 ng/m^3^; floor/carpet stores, 78.25 ng/m^3^; offices, 59.32 ng/m^3^; schools, 36.23 ng/m^3^; daycare centers, 31.80 ng/m^3^; building material markets, 31.17 ng/m^3^; and private homes, 12.51 ng/m^3^ [35]. The results showed that, in indoor air, the concentration of ΣOPFRs ranged from 3.30 to 751.0 ng/m^3^, with a median of 40.23 ng/m^3^, while that in outdoor air was 5.38 ng/m^3^, suggesting that OPFRs are a pollutant that is dominant in indoor air environments compared to the outdoors [35].

**Figure 2:**
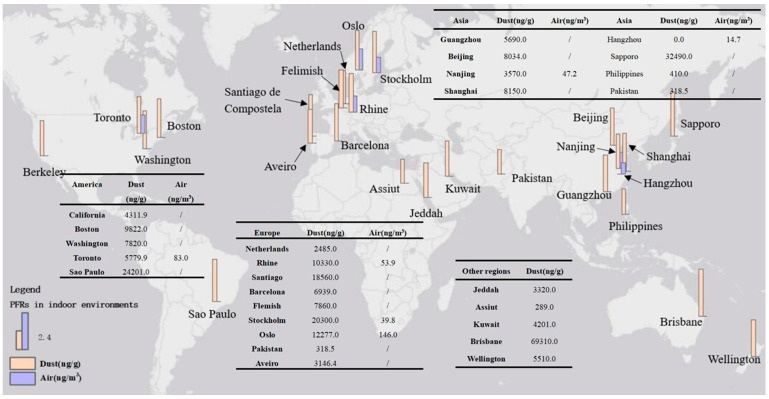
The spatial distributions of the concentrations of selected OPFRs in indoor dust (ng/g) and the locations of the six indoor air testing sites. The concentration unit is ng/m^3^, and the data are all logarithmically processed. “/” represents data not measured in the relevant study.

**Figure 3:**
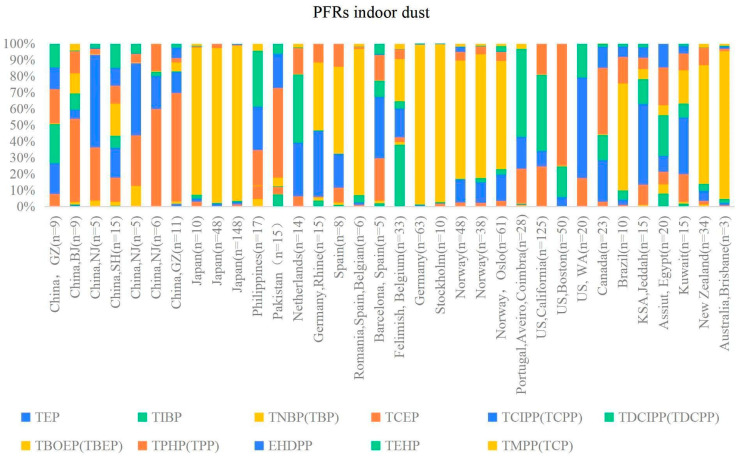
Composition profiles of OPFRs in indoor dust collected from different countries with various sampling numbers.

**Figure 4:**
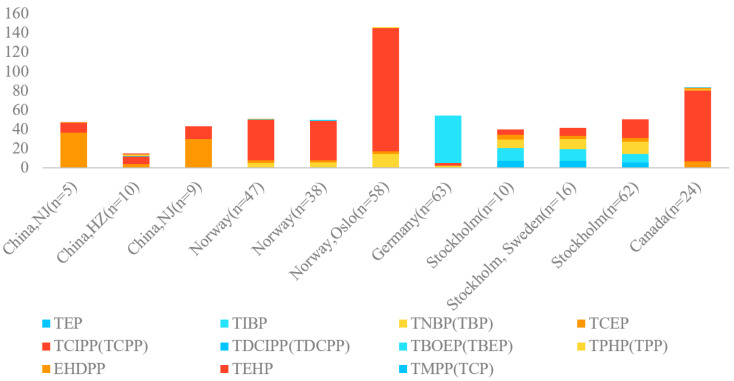
Composition profiles of OPFRs in indoor air collected from different countries with various sampling numbers.

Summaries of the OPFR contents for indoor dust and indoor air from different countries are reported in Table 2 and Table 3, respectively. An overview of the regional levels and trends of the main studied OPFRs is shown in Figure 2, and the compound compositions and patterns of selected OPFRs in indoor dust and indoor air are shown in Figure 3 and Figure 4, respectively. In general, the highest concentration of TBEP appeared in indoor dust, and that of TCPP appeared in indoor air. Dust samples were dominated by OPFRs, including TBEP, TCEP, TPHP, TDCPP, and TEHP. TBEP was found to have the maximum contribution and a global trend of the highest content in indoor dust in this study, which may be explained by its comprehensive range of applicability as a plasticizer; it is often added to floor wax and synthetic rubber[53]. TBEP has a relatively higher molecular weight and a lower vapor pressure, which makes it appear in dust more than in the air. Various factors might impact the OPFR profiles in indoor dust, as their different uses may lead to heterogeneity among countries and regions [54].

In air samples, OPFRs were dominated by TCIPP, TBEP, and TCEP. TCIPP was dominant in Norway (40.8–128 ng/m^3^); Stockholm, Sweden (100 ng/m^3^); and Toronto, Canada (73.6 ng/m^3^). TBEP was dominant in Germany (49 ng/m^3^). And TCEP was dominant in Nanjing, China (29.5–36 ng/m^3^). Compared with TCIPP, the content of TCEP was substantially lower than that of TCIPP in European countries, which is likely due to the restrictions from the European Union on TCEP and the associated rising use of TCIPP as an alternative [55]. The abundance of TCIPP in the air was consistent due to its frequent use and relatively high vapor pressure [56].

Figure 2 shows the worldwide concentration distribution of OPFRs in indoor dust, including 25 countries or cities. The highest concentration of ∑OPFRs was found in Stockholm dust (n = 10), with a median level of 1,646,300 ng/g, followed by Japan (533,250 ng/g, n = 148) and Germany (229,080 ng/g, n = 63). After analysis of variance (ANOVA), there was no significant difference between the data from Sweden and Germany (*p* > 0.05), while the differences between the data from Japan and from Sweden and Germany were significant (*p* < 0.05). Figure 2 also shows the concentration distribution of OPFRs in indoor air at six sites, from which we can see that Oslo, Norway (n = 58), has the highest level (146 ng/m^3^) of ∑OPFRs, followed by Canada (n = 24) with 82.96 ng/m^3^. The difference between the data from Norway and Canada was significant (*p* < 0.05). This indicates that the distribution characteristics of OPFRs in indoor dust present noticeable regional differences.

Based on the chemical structure of OPFRs, they can be divided into three groups: Chloroalkyl phosphates (ΣChlAlkP) including TCEP, TCIPP, and TDCIPP; alkyl phosphates (ΣAlkP) including TIBP, TNBP, TBOEP, and TEHP; and aryl phosphates (ΣAryP) including TPHP and EHDPP. As depicted in Figure 3, in indoor dust, alkyl phosphates presented the highest level in Japan, Spain, Romania, Germany, Stockholm (Sweden), Norway, Brazil, New Zealand, and Australia. Chloroalkyl phosphates presented significant levels in China, the Netherlands, Barcelona (Spain), California (US), Washington (US), KSA (Jeddah), and Kuwait. Aryl phosphates appeared in higher levels in Pakistan, the Philippines, Boston (US), and Canada. These three groups of OPFRs were mainly present in all of the indoor dust studies but with high variability in their concentration levels.

Figure 4 presents the compositions of OPFRs in indoor air, and chloroalkyl phosphates present the highest level in most of the places we studied, except for Germany and Stockholm, where alkyl phosphates are expected to be more dominant. These similarities and differences are probably related to the indoor microenvironments, sources, or materials that each study sampled. Correlated relationships among OPEs between air and dust have been discussed and tested using a partitioning model [12]. This Weschler and Nazaroff partitioning model provides a relationship that can be used to calculate gas-phase concentrations using data from dust, which is expressed as

(1) $$C_{g}=\frac{X_{d}\rho_{d}}{K_{OA}f_{OM}},$$

where *X_d_* is the measured dust concentration (in ng/g), *ρ_d_* is the density of dust (2.0 × 10^6^ g/m^3^), and *f_OM_* is the fraction of organic matter in the dust (0.3, as suggested by Bennett and Furtaw in 2004).

Vykoukalova et al. (2017) have shown that an equilibrium is reached for OPEs with log *K_OA_* values < 12, and under this condition, the partitioning between dust and air is proportional to a chemical’s *K_OA_*. When the log *K_OA_* value is 12, the log dust/air partition coefficient is 3.4 for OPEs, and compared to BFRs, OPEs are more partitioned into the air than in dust [11].

### 3.2. OPFRs from Different Indoor Sources and Materials

Different indoor sources and materials, such as air conditioner (AC) filters, floor coverings, windows, beds, electronic appliances, and building decoration and upholstery materials, were considered for collection and measurement of flame retardants including polybrominated diphenyl ethers (PBDEs), brominated/chlorinated flame retardants (Br/Cl FRs), and organophosphate flame retardants (OPFRs). The concentration and distribution information for major OPFRs in these indoor sources and materials are shown in Table 4. For indoor dust, TBEP was the most abundant OPFR in floors, indicating that its primary source was PVC coverings or floor polishes and waxes. Plastic materials such as computer covers and screens were most likely to be the source of TPP [11]. TCEP was found to be the highest level (94 mg/kg dust) in libraries, indicating that the acoustic ceiling could be a possible source [12]. TCPP is often added to upholstery and is likely emitted from sofas [10]. Consumer products and building materials were collected to measure emissions of OPFRs, and expandable polystyrene (EPS) and extruded polystyrene (XPS) insulating boards were found to be the essential sources of HBCD, while TCPP was observed to be commonly emitted from PU foam products [57]. In Guangzhou, in indoor dust samples from nine bedrooms, ΣPFRs were measured with a level range of 1560–12,600 ng/g in beds; among them, TDCIPP was the predominant component. Moreover, in AC filters, TCEP had the highest concentration, up to 433 ng/g, while in windows, TCIPP had the highest concentration, up to 339 ng/g [12]. In office dust, TPHP was one of the most significant compounds determined in printers and PC tables [25] Car dust collected from car seats was dominated by TDCIPP, with the highest concentration of 1100 μg/g. The primary material of car seats was polyurethane foam, in which TDCIPP and TCIPP were used as additive flame retardants [1]. Regarding building decoration materials in China, OPFRs appeared, in concentrations ordered from high to low, in foam samples, wallpaper, sealing materials, PVC pipes, boards, paints, and wall decoration powder. The Cl-OPFRs had a significantly higher level in foams, while non-Cl-OPFRs were higher in wallpaper (PVC and non-woven) and board samples [5]. TDCIPP, followed by TNBP, had higher median concentrations in the Czech Republic window film samples (Vykoukalova et al., 2017). Electronic equipment also influenced several kinds of organophosphorus flame retardants in houses and cars; for example, Brandsma et al. (2014) found that the concentrations of TPHP and TMPP measured on electronic equipment were much higher than those around electronic equipment in house dust [58]. However, electronics showed a limited contribution to other OPFRs, so the researcher believed that other household materials may have influenced the OPFRs levels in indoor dust. A study in Japan has suggested that the recycling and reuse of electronic products may provide a pathway for OPFRs to go into new products [33].

**Figure 5:**
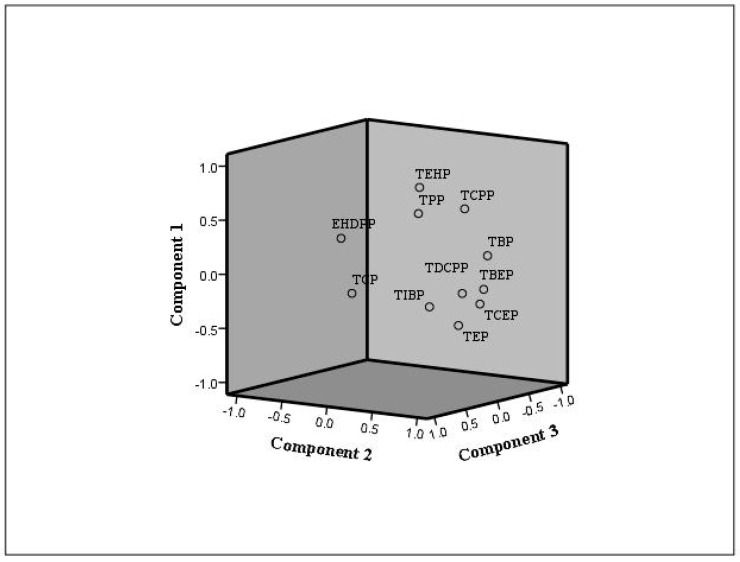
The principal component plot of PFRs in indoor dust environments.

Principle component analysis (PCA) was applied to obtain a further source apportionment of OPFRs in indoor environments using the indoor dust data collected from previous studies [5,10,12,25,56,57] (see Figure 5). The standardized values used as variables were prefixed with Z. There were three main components in the plot, suggesting a similarity of applications of the three groups of OPFRs in the materials. According to the PCA results and emission sources of OPFRs from Table 4, preliminary source resolution for OPFRs in indoor dust can be performed. The first component was mainly contributed to by TBP, TCEP, TBEP, and TDCPP. TBP and TDCPP had an exceedingly high percentage in foam samples normally used as anti-foaming agents. TDCIPP is commonly used as FR inflexible and rigid PUF [50]. TCEP and TBEP are commonly used in building decoration materials such as acoustic ceilings and non-woven wallpaper, especially for PVC floor coverings and floor waxes, where TBEP is mainly used as a plasticizer and floor polish. The second component was mainly comprised of TEHP, TCPP, and TPP. TEHP and TPP are mainly used as flame retardants, plasticizers, and extraction agents, widely added into plastics and processed fibers, such as furniture upholstery, textile carpet padding, isolation materials, and so on. These were probably the main sources or reservoirs of the second component. EHDPP and TCP had remarkably high percentages in the third component. A significant correlation was observed between TCP and EHDPP (r = 0.794, *p* < 0.01), likely as these two aryl phosphates are commonly used in office PCs or printer tables. TCP is also applied widely as a flame retardant in electrical tables and as a plasticizer for automobile car interiors and furniture upholstery [59].

Based on the collected concentration and distribution information of OPFRs in worldwide regions and microenvironments, there were more indoor dust studies than indoor air studies. The difference in research purposes was that dust is more often studied as the potential exposure route for ingestion and dermal contact, while air is usually studied with regard to inhalation in human exposure and the degree of enrichment in different media. As for indoor dust samples, Sweden and Japan had the highest concentrations of ΣOPFRs among the countries studied, especially for floor dust-initiated TBOEP. Thus, it could be predicted that TBOEP was mostly emitted from the ground and was a dominant organophosphorus flame retardant in indoor dust. Similarly, in indoor air samples, we observed that ΣOPFRs had a higher level in Norway and Canada, and TCIPP and TDCIPP were the leading components in indoor air in these countries. They were mostly emitted from PU foam products and upholstery, and they are both chlorine flame retardants. It was predicted that they mostly existed in indoor air due to their volatility in building decoration materials. The molecular weight and vapor pressure of different OPFRs also have an impact on the partition equilibrium in dust and air. The above observations show that contamination from OPFRs, and, thus, their impact on human beings, is higher in indoor dust than in indoor air. This review aims to verify this hypothesis by estimating the human exposure to and toxicity information of different OPFRs in indoor dust and air in the next chapter.

## 4. Health Effect

### 4.1. Human Exposure to OPFRs in Indoor Dust and Air

In indoor environments, there are three main exposure pathways for humans: ingestion, inhalation, and dermal absorption (Thomsen et al., 2001). Some researchers have claimed that FRs are usually associated with human exposure through dust ingestion, while another study suggested that hand-to-mouth contact or dermal absorption might be essential exposure routes for certain Cl-OPFRs such as hydrophilic TCEP and lipophilic TDCIPP [54,59,60]. In Washington State, researchers measured the concentration of ∑Cl-OPFRs with a mean level of 426 ng/m^3^ and found that inhalation and respiration were important routes for the particulate fraction by estimating the inhalation intake of TCPP, which was up to 4540 ng/day for an adult [23]. Fang et al. (2014) have found that Cl-OPFRs have high bioaccessibility and quickly enter the digestive tract and the tissue in the upper respiratory tract [61]. In comparison to the inhalation of FRs, the exposure routes of dietary intake and dust ingestion were more important, but long-term persistent inhalation would make it a potential pathway for the intake of toxic compounds, especially some OPFRs with high vapor pressures. Non-dietary exposure routes such as inhalation and dermal contact have a potential role in causing unintended consequences [62]. In Japan, an exposure assessment of TBOEP in school and house dust was found to be higher than the reference dose, and the highest hazard quotient value of 1.9 in the dust ingested scenario [39]. For Romanian indoor dust, higher exposures to OPFRs were measured with factors below their corresponding reference dose of 50 (in the case of TBEP) and 165 (in the case of TCP) [54]. In the U.K., human dermal absorption of PFRs was studied by using human ex vivo skin and EPISKIN™ models. TCEP, TCIPP, and TDCIPP presented absorbed fractions of 28%, 25%, and 13% of the applied dose (500 ng/cm^2^, finite dose), respectively. Furthermore, the estimated dermal contact exposure to PFRs for toddlers was higher than that for adults in indoor dust [60].

Most of the previous studies have estimated exposure pathways in one type of indoor environment. In addition, the data of factors such as recipient body weight, age range, intake rate, and so on differed between each study, leading to uncertainties in human exposure assessment. To simplify the calculation, a settled body weight is considered for toddlers and adults. In this review, a comprehensive assessment method for human exposure to OPFRs through the three pathways in various indoor microenvironments for adults and toddlers is developed.

#### 4.1.1. Inhalation

Inhalation exposure depends on the concentration of OPFRs in the air, recipient inhalation rate, exposure frequency, and body weight. According to the USEPA (The United States Environmental Protection Agency 1998), the inhalation exposure to air pollutants can be estimated using the following equation:

(2) $$\sum Inhalation=\frac{C_{air}\times IR\times EF}{BW\times 365},\;$$

where *∑Inhalation* is the inhalation exposure to air contaminants (ng/kg/day), *C_air_* is the OPFRs concentrations in indoor air (ng/m^3^), *IR* is the inhalation rate for an adult (20 m^3^/day) or toddler (3.8 m^3^/day), *EF* is the exposure frequency (350 days/year), and *BW* is the body weight for an adult (70 kg) or toddler (20 kg) [60,63,64].

The inhalation exposure to ∑OPFRs in indoor air from different regions ranged from 2.53 to 40 ng/kg bw/day. The high exposure values through air inhalation for adults and toddlers were 3.29, 3.84, 8.08, 35.07, 0.17, 13,42, 0.39, 0.47, and 0.01 ng/kg bw/day and 2.19, 2.55, 5.37, 23.32, 0.11, 8.93, 0.26, 0.31, and 0.01 ng/kg bw/day for TIBP, TNBP, TCEP, TCIPP, TDCIPP, TBOEP, TPHP/TPP, EHDPP, and TMPP/TCP, respectively. Among them, TCIPP, TBOEP, TCEP, and TNBP had relatively higher inhalation exposure values. In this study, the inhalation exposure to ∑OPFRs in Oslo (Norway) and Canada was higher than those estimated in China, Germany, and Stockholm. The estimated exposure to TNBP, TCEP, TCIPP, TDCIPP, TBEP, TPP, and TCP was compared with the reference dose (RfD) of the respective compounds given by USEPA. We found that the inhalation exposure was 3–6 orders of magnitude lower than the RfDs, suggesting an insignificant risk is posed to adults and toddlers through the inhalation of indoor air, according to the data used in this study.

In terms of concentration profiles, Cl-OPFRs showed a higher level in most indoor air places. The total intake of Cl-OPFRs through the inhalation exposure route for adults and toddlers ranged from 0.74 to 35.89 ng/kg bw/day (mean 12.62 ng/kg bw/day) and 0.49 to 23.87 ng/kg bw/day (mean 8.40 ng/kg bw/day), respectively, which were higher than other compounds and indicate that the inhalation exposure pathway appears to be of particular importance and should be taken into consideration in the assessment of these chlorinated organophosphate flame retardants. Among them, inhalation exposure to TCIPP in Norway (mean exposure of 23.13 and 15.38 ng/kg bw/day for adults and toddlers, respectively) and Canada (20.16 and 13.41 ng/kg bw/day for adults and toddlers, respectively) was significantly higher than in other regions and was 2–3 orders of magnitude higher than inhalation exposure for other compounds. TBOEP was the most abundant OPFR, and it showed an estimated high intake via inhalation both for adults and toddlers, especially in Germany (13.42 and 8.93 ng/kg bw/day for adults and toddlers, respectively). These exposure values were approximately 0.089% and 0.060% of the mentioned RfD, indicating that daycare centers contributed to higher TBOEP exposure.

Collectively, the estimated human inhalation exposure to OPFRs by indoor air did not pose an immediate and significant health risk to toddlers and adults, as mentioned above. However, some typical OPFRs, such as TCIPP and TBOEP, showed higher levels of air inhalation in the population in these countries [60,63,64]. Further studies are required to fully characterize the overall human exposure to OPFRs through inhalation exposure pathways.

#### 4.1.2. Ingestion

For indoor dust, ingestion is a significant exposure pathway for flame retardants [42]. Dust ingestion exposure occurs as a result of a migration of OPFRs from indoor materials to dust and subsequent ingestion, which depends on the OPFRs concentration in indoor dust, exposure time, daily dust intake, and body weight. Collected concentrations of OPFRs were used to estimate the exposure of adults and toddlers through indoor dust ingestion. The equation that was used to calculate the total daily intake of OPFRs is as follows:

(3) $$\sum Ingestion=\frac{C_{DI}\times F_{I}\times I_{R}}{BW},$$

where ∑Ingestion is the total daily human exposure to the studied OPFRs through indoor dust ingestion (ng/kg bw/day); *C_DI_* is the concentration of OPFRs in indoor dust (ng/g); *F_I_* is the average fraction of daily time spent in indoor environments; *I_R_* is the mean dust ingestion rate (mg/day), set as 20 and 50 for adults and toddlers, respectively [27]; and BW is the body weight (kg), set as 70 and 20 kg for adults and toddlers, respectively. As detailed information on the time spent in different indoor environments was missing, a person was considered to spend 100% of their time in indoor environments, and so, the figures given here represent the highest exposure scenario of dust ingestion for a worst-case assessment.

The high exposure values via dust ingestion for adults and toddlers were 0.31, 0.29, 1.67, 2.48, 2.86, 145.23, 2.10, 0.45, and 0.27 ng/kg bw/day and 2.75, 2.58, 14.58, 21.73, 25.00, 1270.80, 18.40, 3.98, and 2.38 ng/kg bw/day for TIBP, TNBP, TCEP, TCIPP, TDCIPP, TBOEP, TPHP/TPP, EHDPP, and TMPP/TCP, respectively. Among them, TBOEP, TDCIPP, TCIPP, and TPHP had relatively higher ingestion exposure values. For both groups, the estimated exposure levels for most OPFRs were several orders of magnitude lower than their respective reference doses (RfDs) given by the USEPA.

For Cl-OPFRs, the estimated exposure values from dust ingestion for adults and toddlers ranged from 0.01 to 4.95 ng/kg bw/day (mean: 1.10 ng/kg bw/day) and 0.04 to 43.30 ng/kg bw/day (mean: 9.64 ng/kg bw/day), respectively. Contrasting with the inhalation intake of Cl-OPFRs, the total intake by dust ingestion was estimated lower than intake through the inhalation exposure route for adults but was very close and even higher for toddlers, suggesting that Cl-OPFRs have a higher percentage of the amount reaching respiratory tract and a more significant percentage in toddlers, making them very likely to be exposed to these compounds.

Furthermore, the highest ingestion exposure value was found in TBOEP for toddlers in Japan, which was approximately 8.47% of its respective reference dose. The TBOEP exposures in other countries, such as Germany (64.29 and 562.50 ng/kg bw/day for adults and toddlers, respectively) and Australia (18.00 and 157.50 ng/kg bw/day for adults and toddlers, respectively) were higher than those for any other OPFRs. This might be caused by those higher concentrations of TBOEP, which is normally detected in indoor environments. Therefore, ingestion of indoor dust is suggested as a significant exposure pathway to OPFRs, especially for TBOEP.

After estimating inhalation and ingestion exposures through indoor air and indoor dust, we found that the individual OPFRs presented different major pathways. For example, heavier OPFRs such as TBOEP and TPHP were mainly exposed through dust ingestion, while volatile OPFRs such as TCIPP and TCEP had a major inhalation exposure pathway, similar to the results in the study of Xu et al.[48]. The exposure values of toddlers were lower than those of adults, which can be explained by the lower inhalation rate to body weight ratio in toddlers than adults (0.19 m^3^/day/kg vs. 0.29 m^3^/day/kg). However, in the ingestion exposure scenario, the exposure values of toddlers were higher as, compared to adults, toddlers ingest more dust due to increased hand-to-mouth contact, their frequent close-to-ground behavior, and their lower personal hygienic standards [60].

#### 4.1.3. Dermal Contact

Dermal contact exposure to dust is a result of OPFRs transferring from products to dust through direct contact, which depends on the concentration of OPFRs in the surface dust, exposed surface area, dust amount adhered to the skin, skin absorption fraction, exposure time, and body weight. Studies have measured concentrations of ORFRs from AC filter dust, beds, windows, balconies, and other surface dust to calculate their dermal exposure values [25]. The equation that was used is as follows:

(4) $$\sum Dermal=\frac{C\times SA\times DAS\times F\times EF}{BW},\;\;\;\;\;\;\;\;\;\;\;\;\;\;$$

where ∑Dermal is the dermal exposure value (ng/kg bw/day); *C* is the concentration of OPFRs in indoor dust from AC, beds, or surface dust (ng/g); *SA* is the skin surface area exposed (cm^2^), which is 4615 cm^2^ for an adult and 2564 cm^2^ for a toddler; *DAS* is the dust amount adhered to skin (mg/cm^2^), which is 0.01 mg/cm^2^ for an adult and 0.04 mg/cm^2^ for a toddler; *F* is the fraction absorbed by the skin, which has been reported as 28.3%, 24.7%, and 12.7% for TCEP, TCIPP, and TDCIPP, respectively (as the F values of other OPFRs, including TNBP, TBEP, TPP, TPHP, EHDPP and TMPP, are not available in the literature, the average F of 21.9% was used for TCEP, TCIPP, and TDCIPP); *EF* is the fraction of time spent in houses, offices, or beds; and BW (kg) is the average body weight, set as 70 kg for an adult and 20 kg for a toddler [12]. Due to a lack of data for the fraction of time spent in each indoor microenvironment, both groups were considered to spend all of their time exposed to surface dust, and so, the reported results are the maximum dermal exposure values.

The estimated dermal exposure levels for most OPFRs were on the same order of magnitude as ingestion exposure values, and both were much lower than their RfDs, suggesting that dermal uptake of OPFRs by surface dust is also a significant pathway of human exposure to indoor contaminants. If people are in contact with surface dust all day, the dermal exposure values of OPFRs for adults and toddlers were estimated to be up to 7.632 ng/kg bw/day for TNBP, TCEP, TCIPP, TDCIPP, TBOEP, TPHP/TPP, EHDPP, and TMPP/TCP, which were still lower than their intake through dust ingestion. Among them, the highest estimated dermal absorption exposure was found for TBOEP for both adults and toddlers, followed by dermal absorption estimates for TCIPP, TDCIPP, and TCEP, indicating that dermal dust contact and absorption is still a major exposure pathway due to the high concentrations of these compounds in dust.

The dermal exposure values for most OPFRs, such as TCIPP, TBOEP, and EHDPP, during sleeping, were very close to their dust ingestion exposure values, which indicates that the exposure risks of OPFRs from beds should be given more attention. In addition, the dermal contact values for beds are higher than those for other surfaces. This is probably because people are in contact with beds longer than other household surfaces, such as air conditioners, windows, or balconies. Moreover, the dermal exposure values of toddlers were higher than those of adults. Compared to adults, more dust adheres to the skin of toddlers, and their exposed skin surface area to body weight ratio is higher (65.93 cm^2^/kg vs. 128.20 cm^2^/kg). Therefore, regular and frequent cleaning of surface dust needs to be conducted in order to dilute the concentration of OPFRs in indoor dust.

The combined total exposure to OPFRs by air inhalation, dust ingestion, and dermal contact was generally below the RfD values for both adults and toddlers, with a few notable higher exposures of some typical OPFRs. It should be noted that these exposure values are not necessarily accurate due to the uncertainties in exposure time fractions and potential physiological behaviors, as well as the lack of some specific data values.

### 4.2. Toxicity of Several Typical OPFRs

There is limited knowledge related to the toxicity of OPFRs. Animal toxicity tests have been conducted to evaluate the acute toxicity, including LC_50_, IC_25/50_, or EC_50_, of several OPFRs, and it has been found that algae are highly sensitive to OPFRs, as detailed in Table 5. TPHP has been stated to be the most acute toxic triaryl phosphate to water organisms such as fish, shrimps, and daphnia. Results of animal testing have indicated that TPP has low toxicity, and algae are relatively sensitive to TCP [65]. TCP has been shown to be a possible reproductive toxin and was harmful in a salmonella mutagenicity test [1,66,67]. Both the carcinogenic and non-carcinogenic influences of TCPP and TDCPP have been considered, as they exhibit concentration-dependent neurotoxicity, inhibit DNA synthesis, decrease cell number, and alter neurodifferentiation; meanwhile, another Cl-OPFR, TCEP, was considered to be carcinogenic for animals [1,57,68]. Zebrafish embryos are considered a viable and integrative vertebrate model organism for human hazard assessment due to their high throughput (Sipes et al. 2011). Toxicity testing in zebrafish has suggested that several OPFRs have the potential capacity to affect mammalian biology, and their concentrations inducing toxicity in zebrafish could be in the upper range of potential human exposure [68]. For human beings, TCP and TCEP were considered to have negative effects, and TCEP is a human reproductive toxin, while TPHP has a low impact [1]. TDCIPP and TPP in house dust might be associated with altered hormone levels and decreased semen quality in men [69]. The result mainly covered acute and chronic toxicity tests in aquatic organisms, which were limited by data capacity. Fisk et al. have filled most of these data gaps and predicted ecotoxicity values by using quantitative structure–activity relationships (QSARs) and the ECOSAR program [70].

Human exposure values were calculated through the three pathways for various OPFRs, with respect to adults and toddlers in different regions, and toxicity information was collected for some of them. For indoor air, inhalation exposure to eight OPFRs was evaluated, and the results indicated that this kind of exposure was much lower than the RfDs, suggesting an insignificant risk. However, Cl-OPFRs had relatively higher inhalation exposure values and, so, remained a concern. For indoor dust, two exposure pathways were estimated: Ingestion and dermal contact. These two exposure values were found to have similar orders of magnitude, and both were lower than RfDs; however, among them, TBOEP showed a much higher ingestion exposure than any other OPFR, especially in Japan and for toddlers, which indicated that human exposure to dust was a significant pathway that could pose a risk to the exposed population. The estimated exposure results showed that toddlers were exposed to OPFRs more through indoor dust as they interact more closely with dust than adults. Although all of the estimated exposure values were lower than the reference dose-response values, caution should also be given with the increasing application of OPFRs in indoor environments and the contribution of other human exposure pathways, such as food intake. Exposure to OPFR mixtures could lead to dose-additive effects, even if the individual levels of OPFRs are low [71]).

The toxicity of most of OPFRs is not yet completely understood. The toxicity of individual OPFRs and the toxic effects caused by exposure to OPFR mixtures are still unclear and need to be investigated further [35]. It is worth pondering whether a relationship exists between concentration and human exposure or toxicity information of OPFRs for the sake of conducting an overall evaluation of the environmental effects of these pollutants.

## 5. Discussion and Suggestions for Future Research

As flame retardants—especially OPFRs—are widely distributed in our daily lives, it is important to understand their levels of risk from a global perspective. First, we discussed the development and alteration of various types of flame retardants. Then, we found that most studies on OPFRs in indoor environments have been conducted in Europe. Depending on the difference between countries or cities, microenvironments (e.g., dust or air), and sources (e.g., houses or offices and AC filters, floor, windows or beds, household materials, including floor coverings, wallpapers, and electronic equipment, and so on), OPFRs presented a variety of patterns in their distribution and concentration levels. At present, people spend most of their time in indoor environments and are surrounded by various kinds of OPFRs, which may lead to potential diseases and other health effects. We calculated the estimated human exposure to different OPFRs in adults and toddlers by means of inhalation for indoor air and ingestion and dermal contact for indoor dust. The result demonstrated that most of the exposure values were several orders of magnitude lower than the RfDs, from which we can conclude that there is an insignificant risk for both adults and toddlers with respect to air inhalation, dust ingestion, and dermal contact.

There were some limitations to our review. As we only searched studies in the literature published in the English language, we may have missed some studies related to our study published in other languages. We placed emphasis on OPFRs, but not all kinds of flame retardants, according to the needs of our research. Regarding ingestion and dermal exposure, due to a lack of time distribution information, the estimated exposure values were for the worst-case scenario. Finally, we listed the existing toxicity information for several OPFRs, but data for other chemicals in the group were lacking.

## Figures and Tables

**Table 1:** Compound names, abbreviations (alternate names), CAS numbers, and other properties of OPFRs mentioned in this manuscript.

Name	Abbreviations	Chemical Formula	CAS Number	Molecular Weight	Water Solubility (mg/L) at 25 °C [28,30]	Vapor Pressure (Torr) [16,20]	LogKoa (25 °C) [31]	LogKow (n-Octanol/Water Partition Coefficient) [30]	Identification Ions or *m*/*z* (EI)
Triethyl phosphate	TEP	C_6_H_15_O_4_P	78-40-0	182.15	5 × 10^−5^	1.77 × 10^−1^	2.4	0.8	99,127,155
Tri-phenyl phosphate	TPHP	C_18_H_15_O_4_P	115-86-6	326.29	1.9	6.28 × 10^−6^	7.8	4.59	169.1,325.7
Tripropyl phosphate	TPP	C_9_H_21_O_4_P	513-08-6	224.23	827	2.9 × 10	3.7	2.67	170,233,325
Tri-n-butyl phosphate	TNBP (TBP)	C_12_H_27_O_4_P	126-73-8	266.32	280	1.13 × 10^−3^	5.0	4.00	211,99,155
Tris(2-chloroethyl) phosphate	TCEP	C_6_H_12_Cl_3_O_4_P	115-96-8	285.49	7 × 10^−3^	1.08 × 10^−4^	5.2	1.44	63,143,249
Tris(1-chloro-2-propyl) phosphate	TCIPP (TCPP)	C_9_H_18_Cl_3_O_4_P	13674-84-5	327.56	1.6 × 10^−3^	2.02 × 10^−5^	5.0	2.59	279,201
Tris(1,3-dichloro-2-propyl) phosphate	TDCIPP (TDCPP)	C_9_H_15_Cl_6_O_4_P	13674-87-8	430.90	1.5	4.07 × 10^−8^	7.1	3.80	381,379,191
Tris(2-butoxyethyl) phosphate	TBOEP (TBEP)	C_18_H_39_O_7_P	78-51-3	398.48	1.2 × 10^−3^	2.50 × 10^−8^	9.6	3.65	199,299
2-ethylhexyl diphenyl phosphate	EHDPP	C_20_H_27_O_4_P	1241–94-7	362.40	1.9	2.55 × 10^−6^	11.3	5.37	250.8,77
Tris(2-ethylhexyl) phosphate	TEHP	C_24_H_51_O_4_P	78-42-2	434.64	0.6	2.04 × 10^−6^	8.5	4.22	211.2,99
Tris(methylphenyl) phosphate	TMPP (TCP)	C_21_H_21_O_4_P	1330-78-5	368.37	0.36	6.00 × 10^−7^	8.6	5.11	368,277,165
Tris(isobutyl) phosphate	TIBP	C_12_H_27_O_4_P	126-71-6	266.31	3.72	1.30 × 10^−2^	3.6	3.60	99,155,211

**Table 2:** Concentrations of OPFRs in indoor dust (ng/g) samples from various indoor microenvironments worldwide. Where the median was not available, the geometric mean is given instead.

	Region	Year	Microenvironments	Sample Number	TEP	TIBP	TNBP	TCEP	TCPP	TDCPP	TBEP	TPHP /TPP	EHDPP	TEHP	TMPP /TCP	∑OPFRs	References
Asia	China, Guangzhou	2015	AC filter dust	n = 8	-	-	-	433	272	217	-	160	210	143	-	1435	[35]
			Bed dust	n = 9	-	-	-	65.6	1005	1050	-	172	86.9	368	-	2747.5	
			Floor dust	n = 9	-	-	-	106	251	327	-	281	180	194	-	1339	
			Window dust	n = 9	-	-	-	167	339	95.7	-	199	140	-	-	940.7	
	China, Beijing	2012–2013	Daycare center room floor dust	n = 9	76	32	124	4114	435	791	1010	1116	-	-	336	8034	[14]
	China, Shanghai	2017 *	Living room	n = 15	50	-	200	1200	1500	600	1600	900	900	1200	-	8150	[15]
			Bedroom	n = 15	30	-	300	1000	1600	700	2000	900	900	1400	-	8830	
			Balcony	n = 7	70	-	200	400	2200	500	2000	1200	300	500	-	7370	
	China, Nanjing	2014–2015	Office dust	n = 5	-	-	68	166	238	-	-	30	-	32	-	534	[36]
	China, Nanjing	2014–2015	Office dust	n = 12	-	-	-	1530	910	1330	-	900	-	-	-	4670	[37]
			House dust	n = 6	-	-	-	2140	720	110	-	600	-	-	-	3570	
	China, Guangzhou and Qingyuan	2013–2014	Rural home dust	n = 25	60	-	140	1930	1220	-	200	1090	310	190	-	5140	[38]
			Urban home dust	n = 11	110	-	80	3780	750	-	320	150	360	140	-	5690	
	Japan	2009–2010	Domestic house dust	n = 10	-	-	130	2700	1700	2200	82,000	820	200	-	1200	90,950	[39]
	Japan, Sapporo	2009–2010	Floor dust	n = 48	-	-	-	-	740	-	30880	870	-	-	-	32490	[40]
	Japan	2006	Floor dust	n = 148	-	-	1030	5830	8690	2800	508,320	4510	-	2070	-	533,250	[41]
	Philippines	2008	House dust (malate)	n = 17	-	-	19	34	-	-	-	89	110	140	18	410	[42]
			House dust (payatas)	n = 20	-	-	20	16	-	-	-	71	34	41	7.7	189.7	
	Pakistan	2011	House dust	n = 15	<5	25	<20	15	<20	<5	16.5	175	67	20	-	318.5	[43]
Europe	Netherlands	2013	Floor dust	n = 14	-	-	-	157	815	1051	-	404	-	-	58	2485	[35]
			Surface dust	n = 14	-	-	-	205	3641	3752	-	357	-	-	57	8012	
	Germany, Rhine	2015	Home dust	n = 15	-	380	250	-	4200	-	4300	1200	-	-	-	10,330	[33]
			Office dust	n = 11	420	660	280	-	8500	3200	8600	2900	-	-	340	24,900	
	Spain	2007 *	House dust	n = 8	-	210	250	1700	3900	-	9900	2600	-	-	-	18,560	[44]
	Romania, Spain, Belgium	2012 *	Indoor dust	n = 6	<30	-	190	680	860	3180	63,000	1160	-	-	1140	70,210	[1]
	Spain, Barcelona	2016 *	Home dust	n = 5	-	143	121	1790	2623	706	-	1102	-	454	-	6939	[45]
	Belgium, Flemish	2011 *	House dust	n = 33	<50	2990	130	230	1380	360	2030	500	-	-	240	7860	[1]
	Germany	2011–2012	Daycare center dust	n = 63	-	<300	<300	400	2680	-	225,000	500	-	500	-	229,080	[46]
	Stockholm	2011 *	Private home dust	n = 10	-	1100	300	2100	1600	10,000	4000	1200	-	-	-	20,300	[32]
			Daycare center dust	n = 10	200	700	1200	30,000	3100	9100	1,600,000	1900	-	100	-	1,646,300	
			Workplace dust	n = 10	100	1300	200	6700	19,000	17,000	87,000	5300	-	-	-	136,600	
	Norway	2012	Residential living room dust	n = 48	-	-	55	414	2680	-	13,400	981	617	-	307	18,454	[30]
	Norway	2012	Household dust	n = 38	-	-	54.9	403	2510	501	15,000	1010	-	-	266	19,744.9	[19]
	Norway, Oslo	2016 *	Living room floor dust	n = 61	-	-	-	435	1997	397	8146	722	-	401	179	12,277	[47]
			Living room surface dust	n = 61	-	-	-	455	5241	1130	6796	1228	-	710	334	15,894	
	Portugal, Aveiro and Coimbra	2010–2011	House dust	n = 28	-	-	28	17	-	22	-	662.4	620	1700	97	3146.4	[16]
America	US, California	2000–2001	House dust	n = 125	-	-	-	1067	410.4	2021	-	813.5	-	-	-	4311.9	[48]
	US, Boston	2002–2007	House dust	n = 50	-	-	-	-	572	1890	-	7360	-	-	-	9822	[49]
	US, Longview and Vancouver, WA	2011–2012	House dust	n = 20	-	-	-	1380	4820	1620	-	-	-	-	-	7820	[23]
	Canada, Toronto	2013	House dust	n = 23	-	-	-	181	1470	917	-	2350	754	101	6.9	5779.9	[12]
	Brazil, Sao Paulo State, Araraquara city	2017 *	Apartment dust	n = 10	-	40.1	28.1	237	1870	2250	22,100	3830	1750	549	-	32,654.2	[31]
			House dust	n = 10	-	30.7	12.3	230	771	1370	15,900	3900	1590	397	-	24,201	
			Office dust	n = 5	-	51.7	40.8	237	1820	4480	72,800	6420	2140	500	-	88,489.5	
Africa Middle East	KSA, Jeddah	2016	House floor dust	n = 15	-	-	35	410	1650	500	205	230	220	70	-	3320	[50]
			AC filter dust	n = 15	-	-	10	820	2000	7800	50	600	350	130	-	11,760	
	Egypt, Assiut	2012–2013	House dust	n = 20	-	23	17	22	28	72	18	67	42	-	-	289	[46]
	Kuwait	2011	House dust	n = 15	19	54	58	710	1460	360	855	430	190	65	-	4201	[43]
Oceania	New Zealand, Wellington	2012 *	Indoor dust	n = 34	-	-	80	110	350	230	4020	600	-	-	120	5510	[51]
				n = 16	-	-	70	40	250	110	1550	240	-	-	160	2420	
	Australia, Brisbane	2015	Indoor dust	n = 3	-	<30	140	550	1000	1500	63,000	870	1300	<450	950	69,310	[51]

A year accompanied by * is the publication year, and others are sampling years. “-” means no data.

**Table 3:** Concentrations of OPFRs in indoor air (ng/m3) samples from various indoor microenvironments worldwide. Where the median was not available, the geometric mean is given instead.

	Region	Year	Microenvironments	Sample number	TEP	TIBP	TNBP-	TCEP	TCPP	TDCPP	TBEP	TPHP /TPP	EHDPP	TEHP	TMPP /TCP	∑OPFRs	References
Asia	China, Nanjing	2014–2015	Office air	n = 5	-	-	0.53	36	10	-	-	0.4	-	0.3	-	47.23	[36]
				n = 9	-	-	0.515	29.5	12.5	-	-	0.215	-	0.3	-	43.03	
	China, Hangzhou	2013	Office air	n = 10	-	-	0.47	3.11	7.76	0.63	0.27	1.41	0.22	0.84	-	14.71	[52]
Europe	Norway	2012	Residential living room air	n = 47	-	-	5.09	2.25	42.3	-	0.598	0.258	0.119	-	-	50.615	[30]
	Norway	2012	Household air	n = 38	-	-	5.3	2.4	40.8	0.0753	0.637	0.241	-	-	0.0376	49.4909	[18]
	Norway, Oslo	2016 *	Indoor stationary air	n = 58	-	-	14	3	128	-	-	1	-	-	-	146	[48]
	Germany	2011–2012	Daycare center air	n = 63	-	-	2.2	<2	2.7	-	49	-	-	-	-	53.9	[47]
	Stockholm	2011 *	Private home air	n = 10	7.3	13	9.1	4.8	5.6	-	-	-	-	-	-	39.8	[31]
			Daycare center air	n = 10	1.7	7.2	18	25	8.4	-	84	-	-	-	-	144.3	
			Workplace air	n = 10	6.5	7.3	2.3	10	100	28	5.8	-	-	-	-	159.9	
	Stockholm	2010*	Living room air	n = 16	7	12	11	3.3	8.3	-	-	-	-	-	-	41.6	[31]
	Stockholm	2008	Apartment air	n = 62	5.3	8.9	13	3.9	19	-	-	-	-	-	-	50.1	[17]
America	Canada, Toronto	2013	House air	n = 24	-	-	-	6.35	73.6	0.525	-	0.723	1.71	0.042	0.007	82.957	[12]

A year accompanied by * is the publication year, and others are sampling years. “-” means no data.

**Table 4:** Concentrations of major OPFRs in different indoor sources and building materials.

Different Microenvironments	Major FRs	Concentration Levels	Country	References
PVC floor coverings and floor waxes	TBEP	14–5300 mg/kg dust	Sweden	[10]
Computer screens and TV sets	TPP	3300 mg/m^2^		
Acoustic ceilings	TCEP	0.19–94 mg/kg dust		
Upholstery (sofas)	TCIPP	50 mg/kg dust		
EPS and XPS insulating boards	HBCD	0.1–29 ng/m^2^/h	Europe	[32]
PU foam products	TCIPP	20 ng/m^2^/h–140 μg/m^2^/h		
Bedding Bedroom AC filters	TDCIPP TCEP	1050 ng/g dust 433 ng/g	South China (Guangzhou)	[25]
Bedroom windows	TCIPP	339 ng/g dust		
Office printer table	TPHP	5780 ng/g		
Office PC tables	TPHP	1220 ng/g		
Car seats (PUF)	TDCIPP	1100 μg/g	Netherlands	[58]
	TMPP	380 μg/g		
Wallpaper (PVC) Wallpaper (non-woven) Wallpaper (pure paper)	TEHP TNBP TCIPP	9984 ng/g 102,400 ng/g 177.9 ng/g	China	[5]
Wall decoration powders	TCIPP	5.84 ng/g		
Decoration paints	TDCIPP	156.7 ng/g		
Window films	TCIPP TNBP	566 ng/m^2^ 72.6 ng/m^2^	Czech Republic	[12]

**Table 5:** Toxicity information of several organophosphorus flame retardants.

	Reported 96 h-LC_50_ to Fish (mg/L) [30]	ECOSAR 96 h-LC_50_ to Fish (mg/L) [30]	Acute Toxicity	Long-Term Toxicity
TCIPP	51–84	8.9	Oral: LD_50_ (rat) 500–4200 mg/kg bw Inhalative: LD_50_ (rat) >4.6 mg/L to >17.8 mg/L Dermal: LD_50_ (rabbit) 1230–5000 mg/kg bw [35]	NOEL = 36 mg/kg bw [35]
TDCIPP	1.1	4.7	48 h-EC_50_ (daphnia) 3.8–4.6 mg/L Oral:LC_50_ (rat) 2300 mg/kg Dermal:LC_50_ (rat) >2000 mg/kg [45]	NOEL = 15.3 mg/kg bw per day LOEL = 62 mg/kg perday [28]
TCEP	6.3–250	35		
TBOEP	6.8–24	9.5	15 min-IC_25_/IC_50_ (bacteria) 15.6–500 mg/L 72 h-IC_25_/IC_50_ (algae) 0.18–91 mg/L 96 h-LC_50_/EC_50_ (invertebrates) 7.8–500 mg/L [7]	
TMPP	0.061–0.75	1		
TPHP	0.3–0.66	1	LC_50_ (daphnia) 1.0–1.2 mg/L LC_50_ (rats) 3500–10,800 mg/kg [45]	NOEC = 0.1 mg/L (daphnia)/3500–10,800 mg/kg (rat) [28]
TEHP	>100	0.005	15 min-IC_25_/IC_50_ (bacteria) 0.78–100 mg/L 72 h-IC_25_/IC_50_ (algae) 0.36–182 mg/L 96 h-LC_50_/EC_50_ (invertebrates) 3.13–100 mg/L [7]	

## Data Availability

The data associated with this paper are available on request from the corresponding author.
